# Atlas of ^99m^Tc-RBC scan for gastrointestinal bleeding with emphasis on SPECT/CT imaging: A case series

**DOI:** 10.22038/aojnmb.2025.88577.1637

**Published:** 2026

**Authors:** Mohammad Hadi Samadi, Pegah Sahafi, Ramin Sadeghi

**Affiliations:** Nuclear Medicine Research Center, Mashhad University of Medical Science, Mashhad, Iran

**Keywords:** ^99^mTc-RBC, RBC scan, Nuclear medicine, SPECT/CT A B S T R A C T

## Abstract

Diagnosing gastrointestinal bleeding (GIB) can be especially difficult when endoscopic evaluation do not reveal a clear source. In this report, we describe 20 patients with suspected GIB in whom initial evaluations were inconclusive. All underwent dynamic planar ^99m^Tc-RBC scintigraphy followed by SPECT/CT imaging. This combination proved valuable in either identifying active bleeding sites or clarifying non-bleeding causes of tracer accumulation. The added anatomical detail from SPECT/CT helped distinguish true bleeding from normal physiological activity, vascular landmarks, or postoperative alterations—areas where planar imaging is not enough. In some patients, imperfect red blood cell labeling introduced challenges in image interpretation, occasionally mimicking bleeding. Even so, the fusion of functional and anatomical data improved diagnostic clarity in most cases. This series emphasizes how hybrid nuclear imaging can provide critical insight when other diagnostic methods fail, enabling more accurate localization and better-informed clinical decisions. Our experience supports the broader use of SPECT/CT in evaluating complex or obscure GIB, offering clinicians a noninvasive yet highly informative diagnostic option.

## Introduction

 Gastrointestinal bleeding (GIB) continues to be a clinical problem, particularly for patients with other conditions like chronic kidney failure, cancer, or some type of vascular disorders. Precise bleeding source identification is critical for successful guiding of therapeutic measures. ^99m^Tc-labeled red blood cells (RBC) scintigraphy is a relatively simple noninvasive imaging that assists in the assessment of both overt and occult GIB (1). The possibility of reconciling functional information with precise structural detail in SPECT/CT images has revolutionized this field (2) which can help avoiding false positives such as vascular anomalies and or other anatomical variants, while increasing the chances to detect important low volume or intermittent bleedings (3, 4). In our department, a set of dynamic images, is performed in the anterior and posterior views at 30-second intervals for one hour, immediately after the injection of 20 mCi (740 MBq) of ^99m^Tc-labeled red blood cells (^99m^Tc-RBC). Additionally, 20-minute dynamic images are obtained every 60 minutes. SPECT/CT imaging was performed mainly when a suspicious bleeding site was identified on dynamic planar images, or when the uptake pattern appeared unusual and required further anatomical localization.In the remainder of this article we provided several cases of interest of ^99m^Tc-RBC scan for GIB as an image atlas.

***Case 1***

**Figure 1:**
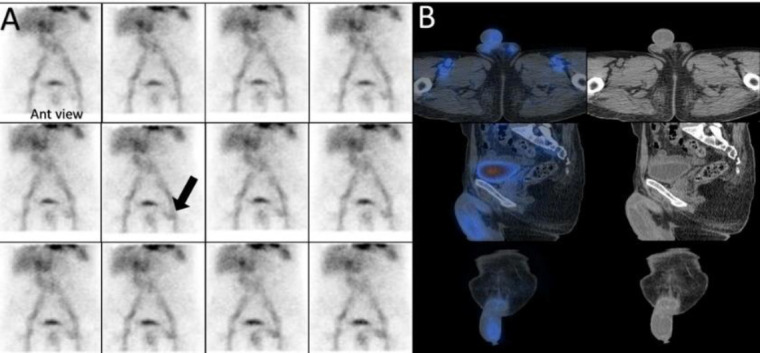
A zone of activity in the left inguinal region fluctuating over time (**arrow-A**) proven to be penile activity on SPECT/CT (**B**)

 A 72-year-old man with a history of progressive weakness, constipation, and melena. His serum hemoglobin level was 7.2 g/dL. A focal zone of radiotracer activity in the left inguinal region that was fluctuating in activity over time, subsequent dynamic images did not demonstrate this zone of activity. Immediate SPECT/CT imaging clarified that the uptake was localized to the penile activity and its crura (Figure 1).

***Case 2***

**Figure 2:**
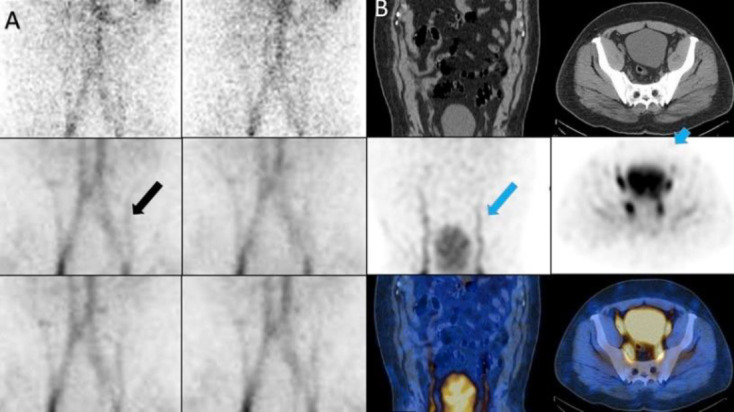
Inferior epigastric veins on planar (**A**) and SPECT/CT (**B**)

 A 47 years old man with history of melena and hematemeisis and Hb: 8.5 g/dl. The dynamic images showed two linear of increased activity in abdomen, symmetrically that SPECT/CT findings was compatible with Inferior epigastric veins (Figure 2).

***Case 3***

**Figure 3:**
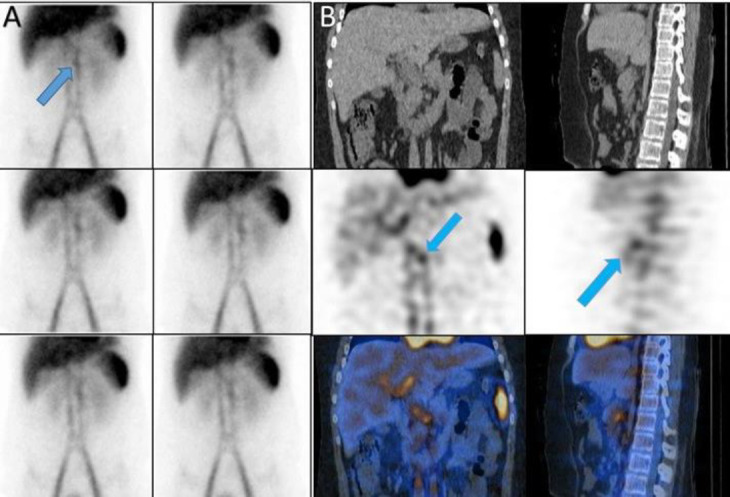
Celiac artery on planar (**A**) and SPECT/CT (**B**)

 A 50 years old man with history of melena and paleness. The planar images showed a focus of 

increased activity in upper abdomen in midline the SPECT/CT confined it to the origin of celiac artery (Figure 3).

***Case 4***

**Figure 4:**
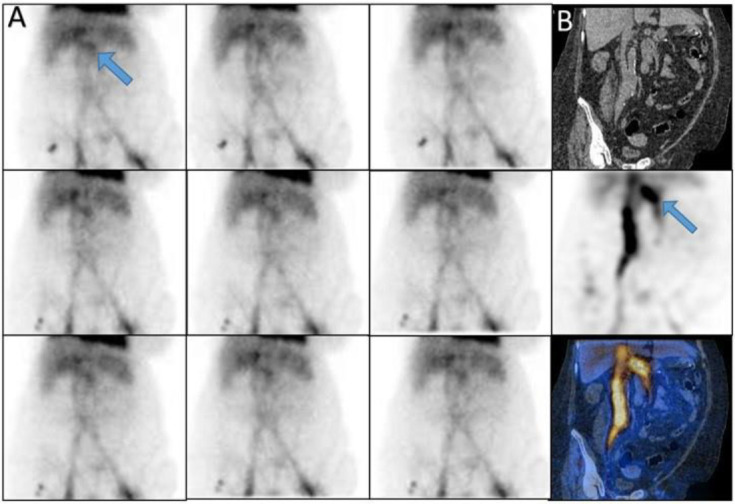
Portal vein on planar (**A**) and SPECT/CT (**B**)

 A 36 years old lady with history of ESRD and transplanted kidney and serum Hb was 8.7 g/dl that endoscopic findings were not convincing for a source of massive rectorrhagia (Figure 4).

***Case 5***

**Figure 5:**
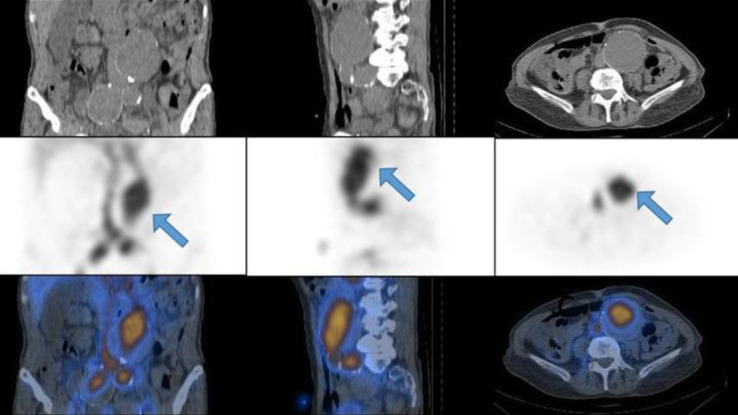
Calcified abdominal aortic aneurysm (CAAA) on SPECT/CT (**arrows**)

 A 78 years old man with history of melena that planar showed zones of increased activity in the 

abdomen that SPECT/CT revealed this findings were compatible with calcified abdominal aortic aneurysm (CAAA) (Figure 5).

***Case 6***

**Figure 6:**
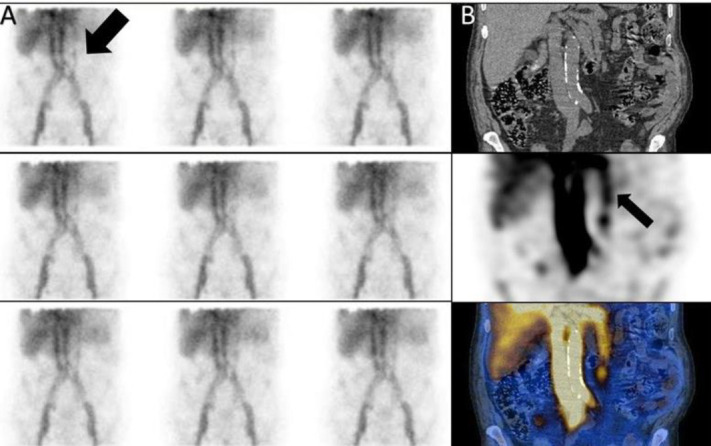
Superior mesenteric vessels on planar (**A**) and SPECT/CT (**B**)

 A 84 years old man with history of AML referred for melena and serum Hb level was 8.9. The planar images showed linear increased activity in the epigastric region which was SPECT/CT showed this activity is consistent with superior mesenteric vessels (Figure 6).

***Case 7***

**Figure 7:**
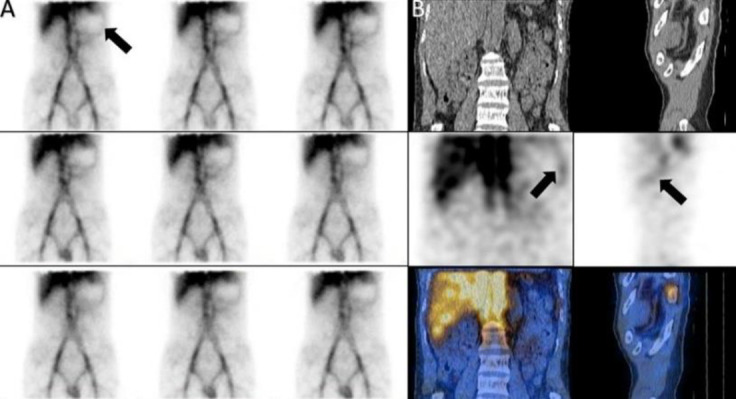
Right gastroepiploic vessels on planar (**A**) and SPECT/CT (**B**)

 A 60 years old man with history of melena. The dynamic scan showed an arcuate shape of increased activity around the photogenic area in the left upper quadrant that SPECT/CT showed this pattern is due to blood pool activity in the Right gastroepiploic vessels (Figure 7).

***Case 8***

**Figure 8:**
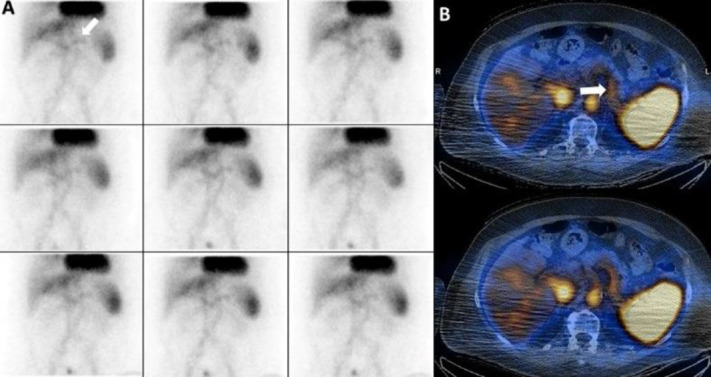
Splenic vessels on planar (**A**) and SPECT/CT (**B**)

 A 70 years old man with history of melena. Dynamic study showed a focus of activity in the epigastric region is compatible with splenic vessels on the SPECT/CT images (Figure 8).

***Case 9***

**Figure 9:**
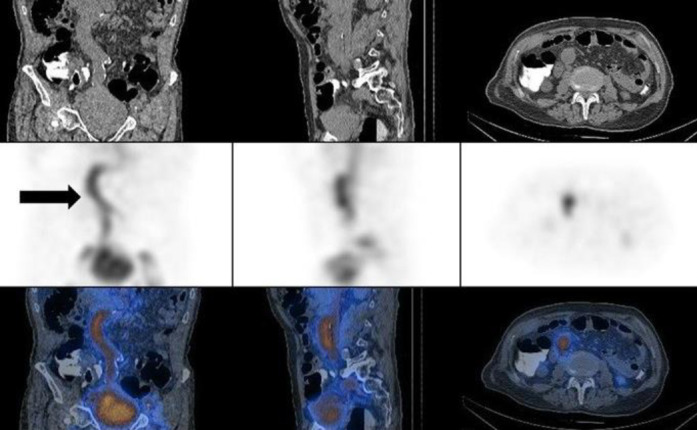
Tortuous inferior vena-cava vein on SPECT/CT images (**arrow**)

 A 81 years old man with history of intermittent rectorrhagia. The planar images showed tortuous activity in the midline of abdomen that SPECT/CT images showed this pattern on inferior vena vena-cava vein (Figure 9).

***Case 10***

**Figure 10:**
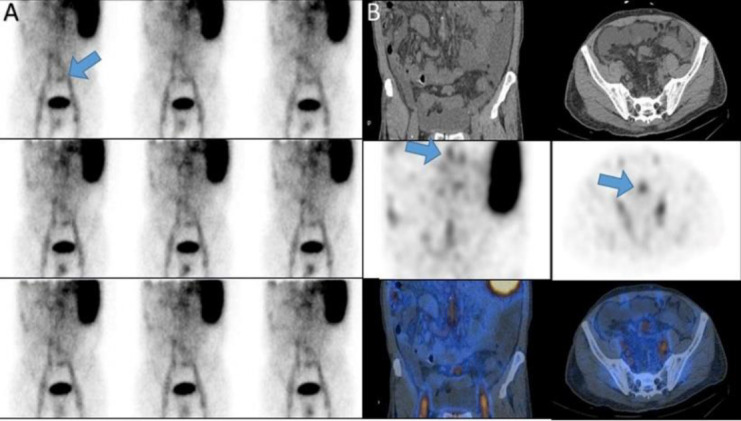
Varicose vessels in a cirrhotic patient on planar (**A**) and SPECT/CT (**B**)

 A 73 years old man with colon cancer and hepatic cirrhosis referred for melena. The dynamic study showed multiple foci of increased activity in the pelvic regions that SPECT/CT showed this foci is consistent with varicose vessels (Figure 10).

***Case 11***

**Figure 11:**
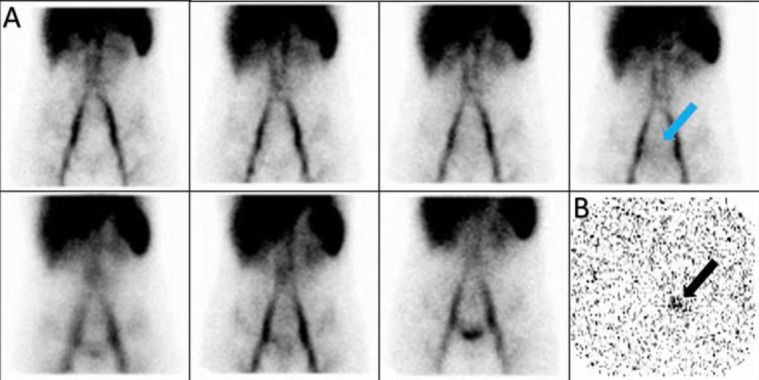
Dynamic images showed a zone of tracer uptake in the pelvis (**arrow A**) proven to be bleeding location by imaging the fecal matter (**B**)

 A 10-year-old girl with rectorrhagia. Dynamic images showed a zone of tracer uptake in the 

pelvis that proven to be bleeding location by imaging the fecal matter (Figure 11).

***Case 12***

**Figure 12:**
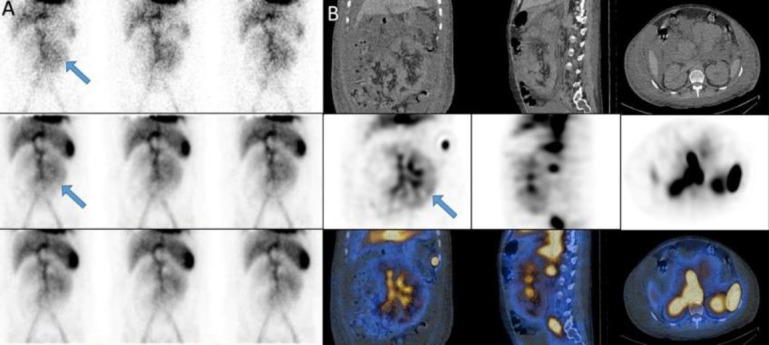
Planar and SPECT/CT images (**arrows A, B**) showed omentum and intestines were displaced and compressed due to ascites

 A 19-year-old lady with lupus nephritis and ascites and rectorrhagia with serum Hb was 5.1g/dl. Planar and SPECT/CT images showed omentum and intestines were displaced and compressed due to ascites (Figure 12).

***Case 13***

**Figure 13:**
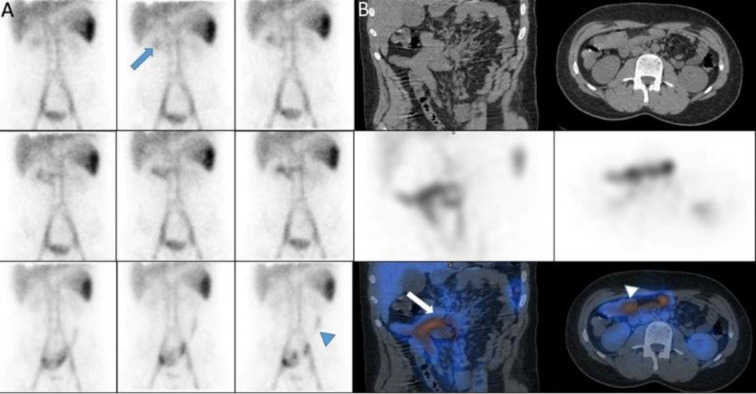
Dynamic (**A**) and SPECT/CT (**B**) images showed the source of bleeding is duodenum with rapid movement to jejunum (**arrowhead**)

 A 17-year-old man with melena. On dynamic images we observed a new zone of increased activity in the right upper abdomen that rapidly moved to the left and then downward, this pattern seems to be due the source of bleeding is duodenum with rapid movement to jejunum. SPECT/CT images confirmed it (Figure 13).

***Case 14***

**Figure 14:**
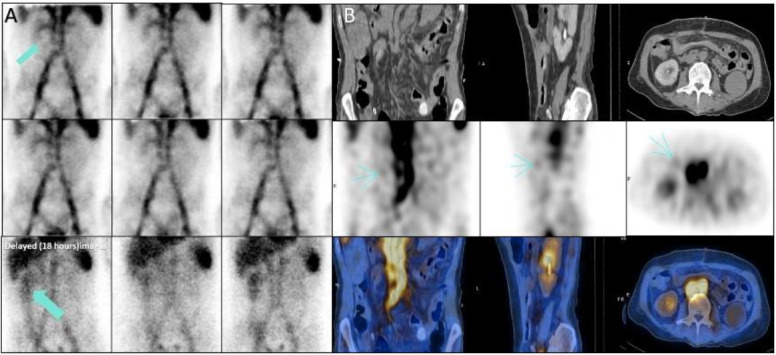
Planar and SPECT/CT images localized the source of bleeding to the anastomotic area (**green arrows A, B**)

 A 59-year-old man with eosinophilic granulomatosis and massive rectorrhagia, who underwent resection of 40cm of jejunum but recurrent massive bleeding led to patient’s referral to our department. The dynamic study localized the small focus of increased activity in the right upper abdomen that was highly suspicious for source of the bleeding, so SPECT/CT was done and showed this focus is compatible with anastomotic area (Figure 14).

***Case 15***

**Figure 15:**
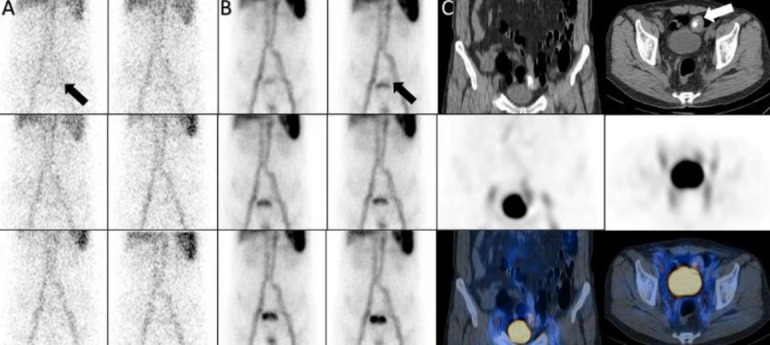
Perfusion and dynamic images showed the suspicious area of increased activity adjacent to the bladder (**arrows in A, B**). SPECT/CT images showed the source of bleeding was benign vascular lesion in the ileum (**arrow, C**)

 A 64-years-old-man with melena from five years ago. Dynamic images showed a suspicious area adjacent to the bladder and SPECT/CT images showed the source of bleeding was calcified lesion in the ileum. Subsequently the lesion was resected and pathological examination showed vascular malformation (Figure 15).

***Case 16***

**Figure 16:**
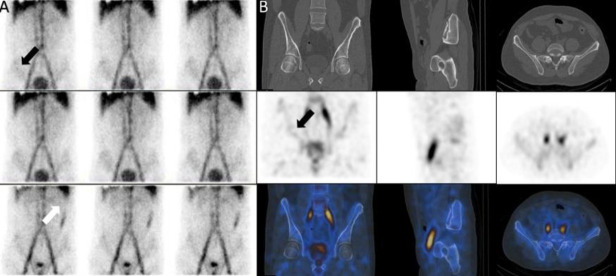
The dynamic scan and SPECT/CT showed activity in bone marrow of iliac alae (**black arrows**). The scan also showed GIB with source of splenic flexure of colon (**white arrow, A**)

 A 28 years old man with history of intermittent rectorrhagia from three months ago and Hb: 7. The dynamic scan and SPECT/CT showed activity in bone marrow of iliac alae due to chronic anemia. The scan also showed GIB with source in the splenic flexure of colon (Figure 16).

***Case 17***

**Figure 17:**
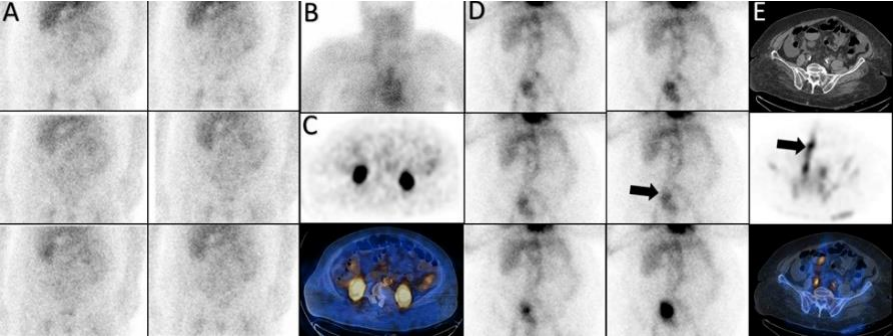
Dynamic images (**A**), cervico-thoracic spot image (**B**) and SPECT/CT images (**C**) in the first day showed sub-optimal with high background activity. In another day, dynamic images and SPECT/CT images showed the source of bleeding was sigmoid colon (**black arrow, D-E**)

 A 73 years old lady with rectorrhagia and repeated blood transfusion. Dynamic and SPECT/CT images in the first day showed sub-optimal scan because large vessels was not well visualized and seems to be due to the very low hematocrit and the scan was repeated using packed cell. Dynamic images and SPECT/CT images showed better quality. Source of bleeding was sigmoid colon (Figure 17).

***Case 18***

**Figure 18:**
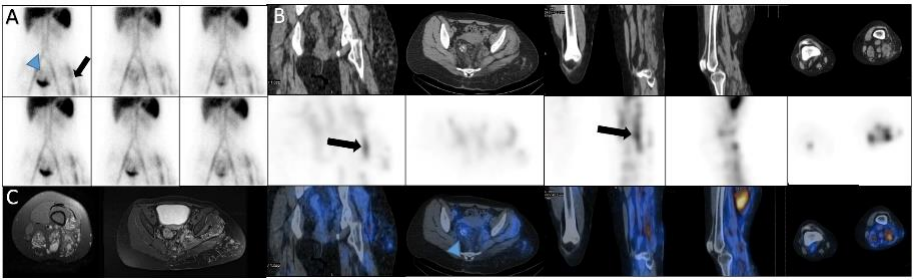
Dynamic (**A**) and SPECT/CT images (**B**) showed soft tissue hemangiomata (**black arrows**). MRI confirmed hemangioma (**C**). Source of bleeding was a rectal hemangioma (**arrow head**)

 A 17-years-old lady with rectorrhagia. MRI findings as well as dynamic and SPECT/CT images showed soft tissue hemangiomata in the left buttock and left thigh. Also, source of bleeding was a rectal hemangioma (Figure 18).

***Case 19***

**Figure 19:**
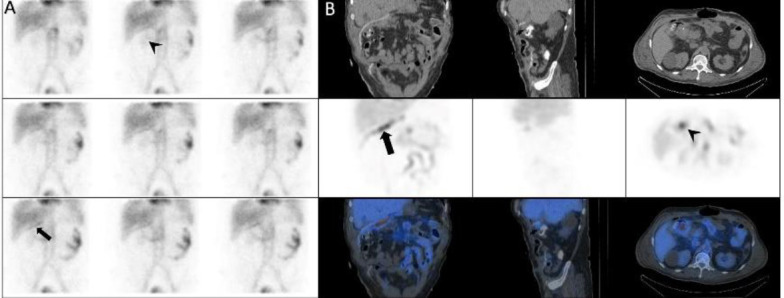
The dynamic (**A**) and SPECT/CT images (**B**) showed activity along the lumen of the surgical drain (**black arrows**). Source of bleeding was duodenal area (**arrowhead**)

 A 50-years-old-man with history of massive rectorrhagia and recent small intestinal resection. Planar images showed linear activity in sub-hepatic region along the lumen of the surgical drain as well as the source of bleeding was duodenal area (Figure 19).

***Case 20***

**Figure 20:**
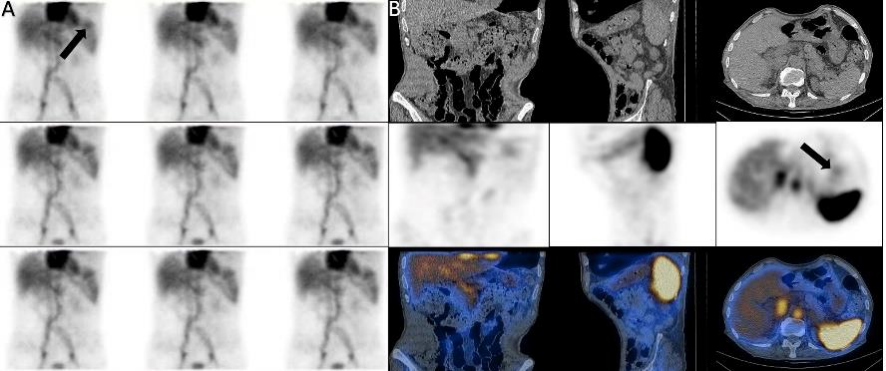
Dynamic (**A**) and SPECT/CT (**B**) showed the source of bleeding in stomach (**arrows**)

 A 72-years-old-man with obscure GIB. Dynamic images showed a large zone of increased activity in the left upper quadrant that the activity moved downward. SPECT/CT showed the source of bleeding in stomach (Figure 20).

## Discussion

 Meta-analyses demonstrate that ^99m^Tc-RBC scintigraphy and CT angiography (CTA) have similar sensitivities for detection of bleeding source in GIB. However, SPECT/CT significantly enhances specificity, by reducing false positives (5, 6). In small bowel bleeding, which was reported that SPECT/CT has a higher diagnostic value than contrast-enhanced multidetector computed tomography (MDCT). This advantage lies in SPECT/CT’s ability to dynamically track tracer movement while precisely localizing it within anatomical structures, making it invaluable in differentiating true intraluminal bleeding from artifacts such as surgical drains or vascular grafts (7). SPECT/CT resolves several common interpretive challenges: Vascular Anomalies: Slow-filling varices or aneurysms may mimic active bleeding on planar imaging. SPECT/CT clarifies these findings through anatomical correlation. For instance, in a cirrhotic patient, SPECT/CT accurately identified portal collaterals previously misinterpreted as colonic haemorrhage (8, 9). Anatomic Variants: Conditions like omental hernias, can lead to tracer pooling due to venous congestion. 

 SPECT/CT identifies such non-bleeding causes, preventing unnecessary interventions (10, 11). Postoperative Changes: Surgical modifications (e.g., stomas or anastomoses) complicate interpretation. SPECT/CT’s three-dimensional mapping helps localize bleeding in these altered anatomies (12). This was evident in a jejunectomy patient with recurrent hemorrhage, where bleeding was pinpointed to the revised bowel segment. Tumoral mass also can cause of false positive interpretation in dynamic images and resolved by SPEC/CT procedure (13).

## Conclusion

^99m^Tc-RBC SPECT/CT has reshaped the approach to gastrointestinal bleeding by offering superior diagnostic accuracy and anatomical resolution. It can resolve false positive issues such as vascular mimicry and altered anatomy.
